# Virtual visits at the Helsinki Head and Neck Center during the COVID-19 pandemic: patient safety incidents and the experiences of patients and staff

**DOI:** 10.1186/s12913-023-09521-5

**Published:** 2023-05-13

**Authors:** Morag Tolvi, Lotta-Maria Oksanen, Lasse Lehtonen, Ahmed Geneid, Pia Männikkö, Hellevi Ruokonen, Anna Majander, Susan Arminen, Leena-Maija Aaltonen

**Affiliations:** 1 Department of Otorhinolaryngology - Head and Neck Surgery, Helsinki University Hospital and University of Helsinki, P.O. Box 263, 00029 HUS Kasarmikatu 11-13Helsinki, FIN Finland; 2 Quality of Care and Patient Safety Department, Head and Neck Center, Helsinki University Hospital, Helsinki, Finland; 3 Department of Phoniatrics, Helsinki University Hospital and University of Helsinki, Helsinki, Finland; 4 Diagnostic Center, HUSLAB, Helsinki University Hospital and University of Helsinki, Helsinki, Finland; 5 Customer Service Department, Head and Neck Center, Helsinki University Hospital, Helsinki, Finland; 6 Department of Oral and Maxillofacial Diseases, Helsinki University Hospital and University of Helsinki, Helsinki, Finland; 7 Department of Ophthalmology, Helsinki University Hospital and University of Helsinki, Helsinki, Finland

**Keywords:** Virtual visits, Patient safety, Patient satisfaction, Staff satisfaction

## Abstract

**Background:**

During the COVID-19 pandemic, health care had to find new ways to care for patients while reducing infection transmission. The role of telemedicine role has grown exponentially.

**Methods:**

A questionnaire on experiences and satisfaction was sent to the staff of the Head and Neck Center of Helsinki University Hospital and to otorhinolaryngology patients treated remotely between March and June 2020. Additionally, patient safety incident reports were examined for incidents involving virtual visits.

**Results:**

Staff (response rate 30.6%, (*n* = 116)) opinions seemed to be quite polarized. In general, staff felt virtual visits were useful for select groups of patients and certain situations, and beneficial in addition to face-to-face visits, not instead of them. Patients (response rate 11.7%, (*n* = 77)) gave positive feedback on virtual visits, with savings in time (average 89 min), distance travelled (average 31.4 km) and travel expenses (average 13.84€).

**Conclusions:**

While telemedicine was implemented during the COVID-19 pandemic to ensure patient treatment, its usefulness after the pandemic must be examined. Evaluation of treatment pathways is critical to ensure that quality of care is upheld while new treatment protocols are introduced. Telemedicine offers the opportunity to save environmental, temporal, and monetary resources. Nonetheless, the appropriate use of telemedicine is essential, and clinicians must be offered the option to examine and treat patients face-to-face.

## Background

During the COVID-19 pandemic, health care had to find ways to care for patients while reducing infection transmission. As the virus replicates in the nasopharynx and many respiratory tract procedures may cause aerosolization, staff working in the head and neck area are thought to be at high risk for infection [1, 2]. Virtual visits have been one option implemented to ensure safety and have been found to be effective, for example, with rhinology patients [3]. Unplanned visits and re-referrals were rare in a two-year follow-up study of otorhinolaryngology virtual visits [4]. Virtual visits reduce waiting times, shorten the length of visits, reduce costs and reduce the carbon footprint of health care by decreasing the need to travel to healthcare facilities [5].

Physician satisfaction with otorhinolaryngology virtual visits was highest with follow-up patients, and when using teleconferencing instead of the telephone in a recent study of 15 respondents [6]. Patient satisfaction with virtual visits has been high – 94% – both in stroke follow-up and otorhinolaryngology (ENT) patients [7, 8]. However, research in the field is still in short supply.

Finland is part of the European Union and follows the General Data Protection Regulation (GDPR, 2016/679) in telemedicine. Finnish national legislation has multiple regulations considering privacy and confidentiality in healthcare. However, there are no specific requirements for the technical systems of remote healthcare in the Finnish legislation, but the supervisory authorities (the National Supervisory Authority for Welfare and Health, as well as the Office of the Data Ombudsman) have enforced some guidelines concerning telemedicine in Finland [9].

Virtual visits were broadly expanded at the Head and Neck Center of Helsinki University Hospital immediately when the COVID-19 pandemic began in Finland in March 2020. The aim of this study was to examine the experiences of staff and patients during virtual visits, and to review patient safety reports. We must examine whether virtual visits are a viable, environmental, and time-saving option in the future as healthcare costs are rising and the resources available are falling.

## Methods

Letters containing a link to the anonymous voluntary online questionnaire, study information, and rights of participants were sent to health professionals of the Head and Neck Center of Helsinki University Hospital. The questionnaire was sent to staff three times to ensure it reached all employees. The Data Management Service of Helsinki University Hospital performed a search for all patients treated remotely by doctors at the Otorhinolaryngology – Head and Neck Surgery Clinic, a tertiary center, March 24—June 30, 2020. This list of patients was validated by cross-checking it with electronic patient records [4]. A visit was classified as virtual if it was conducted remotely by simple telephone conversation or video connection using any digital platform. Patients were excluded if there was no matching physician’s record of the appointment in the patient record system, if the appointment was only rescheduled with no treatment occurring during the visit, or if the patient contact was not real-time and remote [4]. A similar questionnaire and information letter were sent to these patients. To increase the response rate, 100 patients were chosen by random number generator, and were also sent a paper version of the questionnaire and a post-paid return envelope. The questionnaires for patients and staff were created solely for this study.

Helsinki University Hospital uses the national HaiPro Reporting System for Safety Incidents, which is voluntary and anonymous [10, 11]. Patients have also been able to report incidents since May 2021. Safety reports were reviewed for the mention of virtual visits March 1, 2020—December 31, 2021, a follow-up period of 18 months.

Descriptive statistics are presented as means or medians, and numbers with percentages for categorical variables. The relationship between demographic variables and patient and staff opinions was analyzed with cross-tabulation and differences tested with chi-square or Fisher’s exact test. Likelihood to recommend (scale 0–10) was tested with Mann–Whitney U test or Kruskal–Wallis test. A *p*-value of < 0.05 was chosen as significant. Statistics were performed using IBM SPSS Statistics 28 for Windows (IBM Corp., Armonk, NY, USA). Open-ended questions were analyzed using qualitative content analysis.

The study was approved by an independent Ethics Committee (HUS/2242/2020) and by the Research Administration of the hospital (§ 69, HUS/146/2020).

## Results

### Staff questionnaire

The staff questionnaire was sent to a total of 322 residents and 57 nurses and allied health professionals (e.g., opticians, speech therapists, physiotherapists). A response rate of 30.6% was reached (*n* = 116/379): 6 nurses, 44 allied health professionals, and 66 doctors or dentists.

Virtual visits were carried out in numerous different subspecialties of ENT, phoniatrics, ophthalmology, dentistry, and oral maxillofacial surgery (OMS). In addition, 19 speech therapist respondents also treated patients not suffering from head and neck complaints. Twenty-two respondents had no virtual visits. Eighty-two staff members participated in virtual visits by phone, 57 by video and 21 did not answer. Thirty-seven staff members had technical difficulties, 20 did not answer. Most staff (*n* = 73, 62.9%) wished they had a video connection for the virtual visit, eight did not answer.

When asked to grade their likelihood to recommend virtual visits to a colleague, 103 staff members gave an average grade of 7.7 out of 10. Significant factors affecting the success of virtual visits are presented in Tables 1 and 2. Oral disease and OMS specialists were significantly more likely than residents (*p* = 0.014) to recommend virtual visits. Most ophthalmology staff felt conducting virtual visits at home would improve their well-being (borderline significance, *p* = 0.086) (Table 1). Table 3 shows answers on staff well-being.

**Table 1:** Significant factors affecting the success of virtual visits according to ophthalmology staff

	**Time in current profession (*n* = 25)**				**Sex (*n* = 25)**		**Profession (*n* = 25)**	
	** < 5 years (*n* = 3)**	**5—10 years (*n* = 8)**	**11—20 years (*n* = 4)**	** > 20 years (*n* = 10)**	**Male (*n* = 4)**	**Female (*n* = 21)**	**Doctor (*n* = 12)**	**Nurse/Allied health professionals (*n* = 13)**
**Likelihood of recommending to colleague**								
*Median score*	9.00	9.00	9.00	10.00	4.50	10.00	7.00	10.00
*Average score*	9.00	6.67	8.50	7.90	4.75	8.44	5.91	9.64
*p-value*	0.718				0.100		0.012	
**Compared to live visits, virtual visits are**	n (%)	n (%)	n (%)	n (%)	n (%)	n (%)	n (%)	n (%)
*Lighter*	1 (50)	6 (100)	1 (33.3)	8 (88.9)	3 (100)	13 (76.5)	6 (75)	10 (83.3)
*As tiring*	0 (0)	0 (0)	2 (66.7)	1 (11.1)		3 (17.6)	2 (25)	1 (8.3)
*More exhausting*	1 (50)	0 (0)	0 (0)	0 (0)		1 (5.9)		1 (8.3)
*p-value*	0.033				1.000		0.728	
**How often do you manage to completely treat ailments during virtual visits?**								
*Always/most of the time*	1 (50)	3 (50)	2 (66.7)	6 (75)	2 (100)	10 (58.8)	3 (42.9)	9 (75)
*As often as during live visits*	0 (0)	2 (33.3)	1 (33.3)	1 (12.5)		4 (23.5)	1 (14.3)	3 (25)
*Hardly ever/ never*	1 (50)	1 (16.7)	0 (0)	1 (12.5)		3 (17.6)	3 (42.9)	
*p-value*	0.816				1.000		0.077	
**How would conducting virtual visits at home affect work well-being?**								
*Improve*	2 (66.7)	3 (50)	1 (25)	6 (66.7)		12 (63.2)	4 (44.4)	8 (61.5)
*No effect*	1 (33.3)	3 (50)	2 (50)	2 (22.2)	3 (100)	5 (26.3)	4 (44.4)	4 (30.8)
*Worsen*	0 (0)	0 (0)	1 (25)	1 (11.1)		2 (10.5)	1 (11.1)	1 (7.7)
*p-value*	0.754				0.086		0.822

**Table 2:** Significant factors affecting the success of virtual visits according to ENT/phoniatrics staff

	**Time in current profession (*n* = 70)**				**Sex (*n* = 70)**		**Profession (*n* = 70)**	
	** < 5 years (*n* = 15)**	**5—10 years (*n* = 14)**	**11—20 years (*n* = 16)**	** > 20 years (*n* = 25)**	**Male (*n* = 11)**	**Female (*n* = 59)**	**Doctor (*n* = 33)**	**Nurse/Allied health professionals (*n* = 37)**
	n (%)	n (%)	n (%)	n (%)	n (%)	n (%)	n (%)	n (%)
**Compared to live visits, how quickly can you completely treat an ailment during virtual visits?**								
*More quickly*	5 (41.7)	6 (46.2)	4 (25)	9 (39.1)	5 (55.6)	19 (34.5)	16 (55.2)	8 (22.9)
*In the same time*	5 (41.7)	5 (38.5)	4 (25)	8 (34.8)	1 (11.1)	21 (38.2)	7 (24.1)	15 (42.9)
*More slowly*	2 (16.7)	2 (15.4)	8 (50)	6 (26.1)	3 (33.3)	15 (27.3)	6 (20.7)	12 (34.3)
*p-value*	0.522				0.279		0.028	
**Compared to live visits, virtual visits are**								
*Lighter*	8 (61.5)	7 (53.8)	4 (25)	7 (29.2)	7 (77.8)	19 (33.3)	21 (70)	5 (13.9)
*As tiring*	2 (15.4)	2 (15.4)	3 (18.8)	10 (41.7)	0 (0)	17 (29.8)	4 (13.3)	13 (36.1)
*More exhausting*	3 (23.1)	4 (30.8)	9 (56.3)	7 (29.2)	2 (22.2)	21 (36.8)	5 (16.7)	18 (50)
*p-value*	0.154				0.039		0.000	
**How often do you manage to completely treat ailments during virtual visits?**								
*Always/most of the time*	10 (90.9)	10 (76.9)	9 (56.3)	15 (62.5)	5 (62.5)	39 (69.6)	22 (75.9)	22 (62.9)
*As often as during live visits*	1 (9.1)	1 (7.7)	3 (18.8)	1 (4.2)	0 (0)	6 (10.7)	0 (0)	6 (17.1)
*Hardly ever/ never*		2 (15.4)	4 (25)	8 (33.3)	3 (37.5)	11 (19.6)	7 (24.1)	7 (20)
*p-value*	0.212				0.466		0.056	
**How often do you have to book a live visit because ailment could not be treated virtually?**								
*Rarely/ less than half of the time*	9 (100)	8 (66.7)	7 (46.7)	11 (55)	5 (62.5)	30 (62.5)	18 (66.7)	17 (58.6)
*Half of the time*		2 (16.7)	1 (6.7)	1 (5)	1 (12.5)	3 (6.3)	3 (11.1)	1 (3.4)
*More than half/ almost always*		2 (16.7)	7 (46.7)	8 (40)	2 (25)	15 (31.3)	6 (22.2)	11 (37.9)
*p-value*	0.070				0.689		0.317	
**Success of interaction with patients during virtual visits**								
*Excellent/good*	8 (57.1)	10 (76.9)	9 (56.3)	11 (47.8)	4 (40)	34 (60.7)	19 (65.5)	19 (51.4)
*Fair*	6 (42.9)	3 (23.1)	2 (12.5)	9 (39.1)	5 (50)	15 (26.8)	8 (27.6)	12 (32.4)
*Poor/Very poor*	0 (0)	0 (0)	5 (31.3)	3 (13)	1 (10)	7 (12.5)	2 (6.9)	6 (16.2)
*p-value*	0.070				0.329		0.403

**Table 3:** Experiences of 116 staff respondents during virtual visits at the Head and Neck Center of Helsinki University Hospital

	**n (% of respondents)**
**Where would you prefer to do virtual visits?**	
At the hospital	24 (21.6)
At home	21 (18.9)
Does not matter as long as the place is quiet	66 (59.5)
Missing data	5
**Did you have a quiet workspace for virtual visits at the hospital?**	
Yes	83 (85.6)
No	14 (14.4)
Missing data	19
**When taking into account your home situation, is it possible to do virtual visits at home considering confidentiality and a quiet workspace?**	
Yes	93 (83.8)
No	18 (16.2)
Missing data	5
**If you could do virtual visits from home, how would it affect your work well-being?**	
Significantly improve it	29 (25.9)
Slightly improve it	35 (31.3)
It would not affect it	29 (25.9)
Slightly worsen it	7 (6.3)
Significantly worsen it	4 (3.6)
I am not sure	8 (7.1)
Missing data	4

Staff commented on the functionality of virtual visits and were asked to share their opinions in open answer form. Recurrent themes included the importance of patient and ailment selection, and the necessity of one well-functioning digital platform with easy useability. Negative opinions included that virtual visits cannot be used for all patients or for patients who need an interpreter, that a lot of time goes to helping patients with technical problems and connecting to video, and depending on the quality of the internet connection, voice quality may be difficult to evaluate. Virtual visits were useful with young patients as both guardians can participate and staff does not need to go to school or daycare to evaluate the child, saving staff’s time. Staff described virtual visits as patient-friendly: patients belonging to risk groups, working from home, and those living far away do not need to come to the hospital. In addition, virtual visits free up nurses to perform other duties instead of assisting in patient examination. Virtual group rehabilitation and speech therapy worked well and was embraced by the elderly, with only a handful of patients wanting to come to the hospital. Many mentioned the usefulness of virtual visits for follow-ups or as a pre-visit to take patient history. Surprisingly, of those commenting on working from home, many said they would rather work at the hospital due to better ergonomics (e.g., electric adjustable desks) and interaction with co-workers. Some thought the opportunity to work from home would be handy e.g., if one had a mild cold. Some mentioned that open offices made virtual visits impossible due to background noise and lack of confidentiality. Many suggested that virtual visits are a good addition to in-person visits, not in place of them. A few also mentioned the ecological aspect of virtual visits eliminating the need to travel to the hospital, thus reducing health care’s carbon footprint.

### Patient questionnaire

**Fig. 1:**
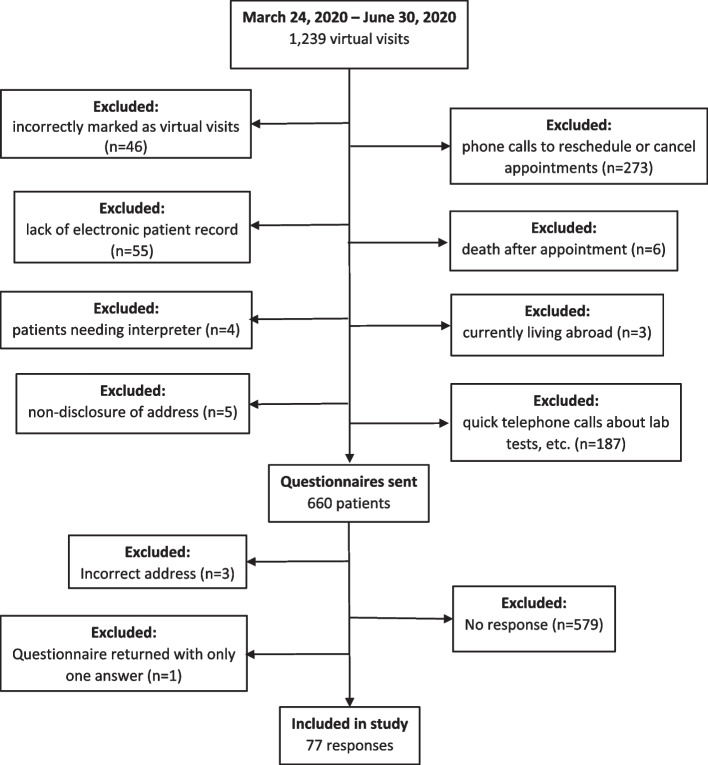
Flow chart of study participants

In this study, 660 virtual visit patients were sent a questionnaire and study information letter (Fig. 1). Seventy-seven responses were received (response rate 11.7%; mean age 53.2 years, median 57.7 years) (Table 4). Most (*n* = 48, 63.2%) used less than 30 min (mean 29.7 min, median 23.0 min) to participate in virtual visits. When asked how much time they would have had to use if they had had a face-to-face appointment at the hospital, most (*n* = 23, 29.9%) answered 2–4 h (mean 118.7 min). Few (*n* = 19/75, 25.3%) wished they had a video connection for the visit. Patients travelled a mean of 31.4 km and saved a mean of 13.84€. When asked their likelihood to recommend virtual visits to a friend, 77 patients gave an average grade of 6.8 (scale 0–10). No significant relationship between likelihood to recommend and age, sex, ailment, distance, money saved, and mode of transport was found. The further away from the hospital the patient lived the more likely the patient was to recommend virtual visits (borderline significance, *p* = 0.050). No significant relationship existed between patient-staff interaction and age, sex, ailment, distance, and money saved. Patients who would have walked or bicycled to the hospital ranked patient-staff interaction as poor or very poor significantly more often than those using other transport (*p* = 0.013). Patients with a shorter distance to the hospital ranked staff interaction more poorly (borderline significance, *p* = 0.050).

**Table 4:** Demographics and experiences of 77 patient respondents during virtual visits at the Head and Neck Center of Helsinki University Hospital

	**n (% of respondents)**
**Sex**	
*Male*	36 (46.8)
*Female*	41 (53.2)
**Age group (years old)**	
*8–16*	1 (1.3)
*17–23*	5 (6.5)
*24–39*	15 (19.5)
*40–55*	14 (18.2)
*56–69*	27 (35.1)
*70–74*	8 (10.4)
*75–92*	7 (9.1)
How would you have travelled to the hospital?*	
*Walk or bicycle*	7 (9.1)
*Bus*	24 (31.2)
*Tram*	24 (31.2)
*Train*	14 (18.2)
*Metro*	11 (14.3)
*Taxi*	5 (6.5)
*By car*	40 (51.9)
**Subspecialty of complaint^**	
*Otology*	10 (13.0)
*Rhinology*	37 (48.1)
*Laryngology*	7 (9.1)
*Tumor*	11 (14.3)
*Tonsils*	11 (14.3)
*Salivary glands*	1 (1.3)
*Voice*	4 (5.2)
*Other*	11 (14.3)
*Hearing*	4 (5.2)
**Did you have to miss work because of your virtual visit?**	
*No, I stopped working for the time of the virtual visit*	29 (37.7)
*No, I do not work*	31 (40.3)
*No, I did not have work at the time of the virtual visit*	14 (18.2)
*Yes. It is not possible to have a quiet phone conversation/video chat at work*	3 (3.9)
**Would you rather have taken care of your treatment**	
*By virtual visit?*	24 (31.2)
*With a regular visit at the hospital?*	35 (45.5)
*I don't know*	18 (23.4)

*33 patients (42.9%) would have used 2 or more modes of transport

*^15 patients (19.5%) marked more than one complaint*

### Patient safety incidents

From March 1, 2020 until December 31, 2021 1,365 patient safety reports were submitted by Head and Neck Center staff; only two were related to virtual visits—both occurring during 2021. One was a misunderstanding, in which the patient did not attend laboratory testing before the virtual visit due to unclear instructions. The other incident occurred years earlier but came to light during a virtual visit when the treating physician noticed the patient had not been operated on as had been planned. In other words, no patient safety reports were filed because of an event directly occurring during a virtual visit.

## Discussion

Staff opinions seemed to be quite polarized – both vehemently for and against virtual visits. In general, staff felt virtual visits were useful for certain situations and patient groups, and beneficial in addition to face-to-face visits, not instead of them. Virtual visits brought savings in both time and money, in addition to reducing the carbon footprint of health care.

Most patients and staff felt ailments were completely treated during virtual visits, and staff rarely needed to reserve a face-to-face visit for patients due to inability to treat during virtual visits. These patients rarely had unplanned visits after virtual visits and most needed no face-to-face follow-ups in a previous 2-year follow-up study by our group [4]. Patient selection for these virtual visits seems apt.

Staff felt virtual visits took the same amount, or less, time. Virtual visits saved patients’ time dramatically (mean 29.7 min vs. 118.9 min). This temporal savings is also compelling to employers, which is often forgotten when calculating the cost of health care. While savings in travel costs were small, the fee billed from patients for virtual visits is also smaller than for face-to-face visits, thus increasing patient savings. In addition, one-third of patients said they would have had to take time off work if the visit had been at the hospital, consequently increasing patient savings and socioeconomic productivity. More than half of patients would have travelled by car, taxi, or bus i.e., means of transport that produce carbon emissions. Patients on average would have travelled 31.4 km. By replacing visits, which do not necessarily need to be face-to-face, with virtual visits, carbon emissions can be greatly reduced and even a small reduction is significant to the environment.

The success of the standardized virtual visit protocol for tonsil patients at our clinic prompted the development of a digital treatment pathway, which greatly reduced the time required for staff to treat these patients, cutting costs even more, and leading to the cessation of tonsil virtual visits [12]. Consequently, virtual visits can be a testing ground for future development of digital patient services.

Dedicated space for virtual visits is a must to ensure confidentiality and allow a calm environment for patient treatment without interruptions. Patient trust may be lost if a patient hears or sees others or feels staff is unable to concentrate on their ailment. Patients with hearing loss may have trouble participating in virtual visits if background noise drowns out the voice of their clinician or the connection is not clear. Evaluation of voice problems is also hampered if the patient’s voice cannot be properly heard, and vice versa, during speech therapy.

Working from home via virtual visit, even occasionally, was important to some to improve their work wellness and could provide an excellent alternative to sick leaves for ailments that do not necessarily impair the ability to work. Virtual visits could possibly decrease infection transmission in hospitals between patients and staff, decreasing the staff’s risk of illness. Most staff members felt virtual visits were as or less exhausting than face-to-face visits, making virtual visits a good way to lighten patient lists and free up nursing and room resources.

One possible downside of virtual visits is their impact on staff training. Training of junior staff members during virtual visits is rather complex, depending on the platform used for the visit. If the telephone is used, it may be confusing to the patient as to who is talking and what their role is. If using a video or digital platform, the senior and junior members of staff must be in the same room, therefore limiting telecommuting.

Surprisingly, only a minority of patients, but most staff, wished they had a video connection. The inability to gauge patient reaction and observe body language and physical features was seen by staff as a weakness of virtual visits without a video connection. A previous study found video connectivity, audio/visual definition, and ease of use to be important factors in patient satisfaction during virtual visits [13], which were mentioned as important to staff satisfaction in this study.

Finland has always been at the forefront of mobile services and digitalization: 97% of Finns have used the internet in the last three months compared to 66% worldwide [14]. Of 16–74-year-old Finns, 96% have smart phones [15]. Finns’ familiarity and comfort with digital services probably helped with their acclimation to virtual visits. In countries with less digital nativity, care should be taken to ensure patients have access to the tools necessary for virtual visits. For patients without this equipment, other forms of treatment must be available to insure equity of care.

Whenever new practices are introduced in medicine, safety must be evaluated. The hospital district requires units to perform risk analysis and draw up a self-monitoring plan to guarantee patient safety and quality of care before starting virtual visits. No patient safety incidents were filed connected to events occurring during virtual visits. While the HaiPro system is voluntary, it does give us an idea of safety. No missed diagnoses were found upon chart review for the patients in this study [4]. However, the potential for missed diagnoses must be remembered when conducting and planning telemedicine services. A standardized protocol for the most common ailments being treated, including red flag symptoms, is one approach to prevention. In addition, the availability of live visits must be secured so patients can also be examined if a red flag comes up during a virtual visit or if staff otherwise feels it is essential to treatment. Patients should be able to opt out of virtual visits if they are not comfortable with telemedicine. Patients must also be clearly informed as to how their treatment will continue after the virtual visit and what to do if they do not receive the next appointment or referral. Involving and activating patients in their treatment is crucial for the success of telemedicine. This is one example of how telemedicine can be used to monitor treatment between live visits.

Limitations of the study included its retrospective nature and small sample size in the patient group. The patient response rate is very low and therefore one cannot make any generalizations about the patient viewpoint of the service. Rather the findings can be an indication as to where further research can be undertaken. Readers must take this into account when assessing the data in the paper. As questionnaires were sent to patients sometime after their virtual visit, their answers may not be completely accurate due to recall bias. We also did not approach patients from other head and neck center specialties by questionnaire. Patients with eye or oral ailments may have felt differently than ENT patients. However, staff respondents comprised a good mix of professions, levels of experience and subspecialties. Non-responder bias is also present, as with all studies involving questionnaires. Nevertheless, the distribution of responders and non-responders into age groups and ailments is very similar, thus ensuring a representative sample.

## Conclusions

While telemedicine was implemented during the COVID-19 pandemic to ensure patient treatment, its usefulness after the pandemic must be examined. This is an opportunity to advance leaps and bounds in how we offer services to patients. It is important to evaluate treatment pathways and ensure that the quality of care is upheld while new treatment protocols are introduced.

## Data Availability

The datasets used and analyzed during the current study are available from the corresponding author on reasonable request.

## Abbreviations

COVID-19: Coronavirus disease
ENT: Ear, nose and throat
GDPR: General Data Protection Regulation
OMS: Oral and maxillofacial surgery
